# Is referral of postsurgical colorectal cancer survivors to cardiac rehabilitation feasible and acceptable? A pragmatic pilot randomised controlled trial with embedded qualitative study

**DOI:** 10.1136/bmjopen-2015-009284

**Published:** 2016-01-04

**Authors:** Gill Hubbard, Richard Adams, Anna Campbell, Lisa Kidd, Stephen J Leslie, Julie Munro, Angus Watson

**Affiliations:** 1 School of Health Sciences, University of Stirling, Inverness, UK; 2 Institute of Cancer & Genetics, Cardiff University School of Medicine, Velindre Hospital, Cardiff, UK; 3 Faculty of Life Science, Sport and Social Sciences, Edinburgh Napier University, Edinburgh, UK; 4 Faculty of Health and Social Care, Robert Gordon University Aberdeen, UK; 5 NHS Highland, Cardiac Unit, Raigmore Hospital, Inverness, UK; 6 NHS Highland, Colorectal Surgery, Raigmore Hospital, Inverness, UK

**Keywords:** REHABILITATION MEDICINE

## Abstract

**Objectives:**

(1) Assess whether cardiac rehabilitation (CR) is a feasible and acceptable model of rehabilitation for postsurgical colorectal cancer (CRC) survivors, (2) evaluate trial procedures. This article reports the results of the first objective.

**Design and setting:**

A pragmatic pilot randomised controlled trial with embedded qualitative study was conducted in 3 UK hospitals with CR facilities. Descriptive statistics were used to summarise trial parameters indicative of intervention feasibility and acceptability. Interviews and focus groups were conducted and data analysed thematically.

**Participants:**

People with CRC were considered for inclusion in the trial if they were ≥18 years old, diagnosed with primary CRC and in the recovery period postsurgery (they could still be receiving adjuvant therapy). 31% (n=41) of all eligible CRC survivors consented to participate in the trial. 22 of these CRC survivors, and 8 people with cardiovascular disease (CVD), 5 CRC nurses and 6 CR clinicians participated in the qualitative study.

**Intervention:**

Referral of postsurgical CRC survivors to weekly CR exercise classes and information sessions. Classes included CRC survivors and people with CVD. CR nurses and physiotherapists were given training about cancer and exercise.

**Results:**

Barriers to CR were protracted recoveries from surgery, ongoing treatments and poor mobility. No adverse events were reported during the trial, suggesting that CR is safe. 62% of participants completed the intervention as per protocol and had high levels of attendance. 20 health professionals attended the cancer and exercise training course, rating it as excellent. Participants perceived that CR increased CRC survivors’ confidence and motivation to exercise, and offered peer support. CR professionals were concerned about CR capacity to accommodate cancer survivors and their ability to provide psychosocial support to this group of patients.

**Conclusions:**

CR is feasible and acceptable for postsurgical CRC survivors. A large-scale effectiveness trial of the intervention should be conducted.

**Trial registration number:**

ISRCTN63510637.

**Strengths and limitations of this study**

- The generalisability of the results is limited because the pilot was small-scale involving only 3 out of a possible 312 cardiac rehabilitation (CR) programmes throughout the UK and involving only small numbers of CR and colorectal cancer (CRC) clinicians and people with CRC and cardiovascular disease.
- People with CRC who agreed to participate in this study may be particularly keen to increase their level of physical activity, which means that the findings from may not be applicable to people with CRC who are likely to be less interested in being physically active to aid their recovery and reduce risk of recurrence.
- The interviews were conducted by the investigators involved in collecting baseline and follow-up measures from CRC survivors, which may have influenced the extent to which participants were willing to be critical.

## Background

There are approximately 28 million people living with and beyond a cancer diagnosis in the world.^1^ Colorectal cancer (CRC) is the fourth most common cancer in the UK with approximately 244 000 CRC survivors.^2^ The American Cancer Society and the World Cancer Research Fund recommend that cancer survivors would benefit from following lifestyle recommendations for secondary cancer risk reduction (eg, taking a nutrient-dense diet, increasing levels of physical activity, smoking cessation, alcohol reduction and avoidance of excess body fat).^3^ ^4^ There is strong evidence that CRC survivors would benefit from meeting recommendations for physical activity (ie, 150 min per week of moderate intensity physical activity); these recommendations are derived from epidemiological observations of relationships between physical activity and cancer survival,^5–7^ and evidence of cause and effect derived from randomised controlled trials (RCTs) about the benefits of physical activity on psychosocial domains, such as quality of life, fatigue, anxiety and depression.^8–10^

Evidence suggests, however, that most CRC survivors are not meeting the recommended level of physical activity.^11–16^ Furthermore, the provision of rehabilitation to promote and support behaviour change among cancer survivors is not standard practice in the UK or indeed, elsewhere.^17^ Integrating rehabilitation into standardised models of care to support cancer survivors to increase their engagement in physical activity, as well as how best to provide this model of care, remains a key public health challenge.

Cardiac rehabilitation (CR) may be an appropriate model to aid recovery from cancer and associated treatments^18^ because (1) physical activity is the cornerstone of CR, (2) CR is evidence-based and draws on theories of behaviour change, (3) CR multiprofessional teams have the expertise required to monitor physical activity to a wide variety of patients including cancer survivors, and (4) CR is widely available throughout the UK and is considered a standard practice in the care of cardiac patients.^19–22^

An aim of the CRIB (Cardiac Rehabilitation In Bowel cancer) study was to assess whether CR is a feasible and acceptable model of rehabilitation to aid the recovery of CRC survivors (ie, examine intervention implementation potential). As far as we know, this study is novel in that it aims to test CR for a different (ie, not people with cardiovascular disease (CVD)) patient group (ie, CRC survivors). We undertook a pragmatic pilot RCT, which included an embedded qualitative study. A description of the study protocol has been published.^23^ In this article, we describe and report data that directly addresses the feasibility and acceptability of the intervention (ie, CR) for postsurgical CRC survivors. The study consent rate can be used as a proxy for likely demand of CR if it was to be implemented in practice. Reasons for declining to participate provides an indication of barriers to up-take of CR by CRC survivors, the number of adverse events provides an indication of the safety of CR for this group of cancer survivors and intervention adherence can be used to estimate likely use of CR by CRC survivors. Thus, in this article, we report these trial parameters. The results of the evaluation of cancer and exercise training and the embedded qualitative study about people's (CRC survivors, people with CVD, cancer and cardiac clinicians) perceptions of CR for CRC survivors are also reported. We aim to describe and report data that directly address the feasibility and acceptability of trial procedures, as opposed to the intervention, separately.

## Methods

### Trial methods

**Figure 1:**
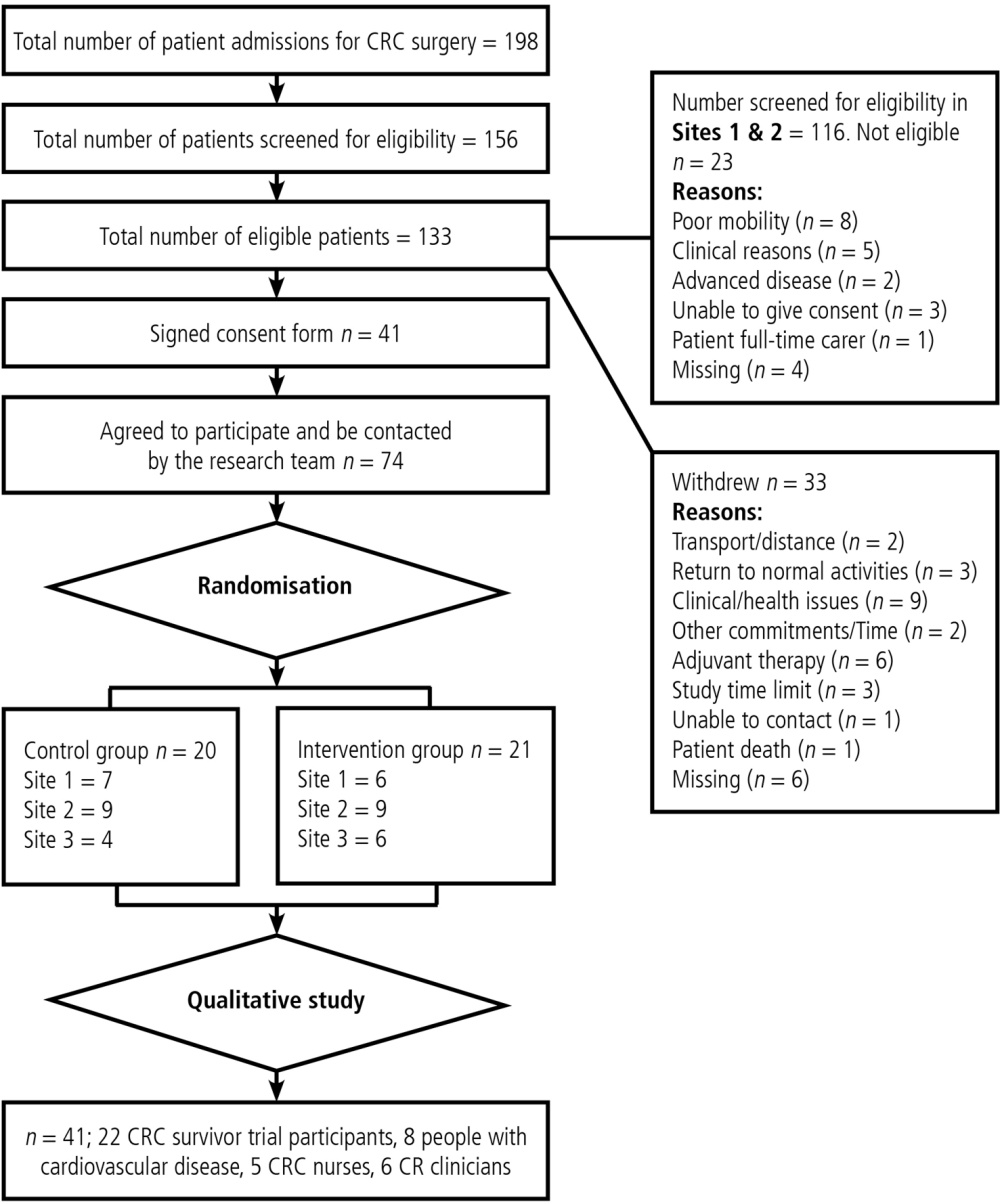
Flow chart (CR,cardiac rehabilitation; CRC, colorectal cancer).

Figure 1 shows participant flow through the study.

#### Participants

People with CRC were recruited from three UK hospitals with CR facilities and considered for inclusion if they were 18 years old and over and had been diagnosed with primary CRC and were in the recovery period postsurgery (they could still be receiving adjuvant therapy). People with CRC were excluded if they had advanced disease, failed clinical/risk assessment for rehabilitation and were deemed unsafe to participate in exercise classes, had severe cognitive impairment or were unable to communicate in English since this is the language used in CR in the UK.

#### Recruitment

A CRC nurse assessed people admitted for surgery for CRC to determine their eligibility for the study; those eligible were given a study information sheet. After discharge from hospital, an investigator contacted people by telephone to confirm willingness to participate. If the person was willing and ready to attend CR, a mutually convenient time for the person to meet with the investigator was arranged where eligibility was confirmed and written consent was obtained. Consented participants had baseline measures taken and were then randomised to either the intervention or control group. If the person decided not to participate in the study, a reason for declining to participate was recorded.

#### Randomisation

Randomisation of individual participants to a particular treatment arm was undertaken using an automated online randomisation system.

#### Treatment group allocation

*Usual care*: Patients were given a booklet by Bowel Cancer UK (a cancer charity)—‘Staying healthy after bowel cancer’.

*Intervention*: Patients were informed they would be referred to CR. One of the key reasons why CR was chosen for patients with CRC is that physical activity (our proposed primary outcome for the full trial) is the cornerstone of CR. The investigator completed a referral form and sent it on to the CR service. A member of the cardiac multidisciplinary team (eg, cardiac physiotherapist or nurse) then contacted the patient and invited them to attend a CR clinical/risk stratification assessment to determine whether the patient was able to safely exercise from a cardiac clinical perspective. Patients who were deemed safe to exercise were then given a date to start CR, which comprised exercise classes and cardiac-specific education sessions. We have used the Template for Intervention Description and Replication (TIDieR)^24^ to describe the CR intervention and table 1 compares the three sites, highlighting key differences.

**Table 1:** Comparison of three cardiac rehabilitation sites (TIDieR)

Intervention component	Site 1	Site 2	Site 3
WHAT—materials	BHF booklets Local activities Home exercises	BHF booklets CHSS resources Local activities Leaflet on exertion and pacing Home exercise cards	BHF booklets An MI and surgery leaflet about recovery Home exercise sheets, if appropriate
WHAT—procedures	1:1 initial assessment ISWT *Class* (15 min warm up; 20 min stations (2×10 min); 15 min cool down) Stretching and relaxation Weekly information seminars	1:1 initial assessment *Class* (15 min warm up; 30 min stations; 10 min cool down) Followed by stretching/Tai Chi Weekly information seminars	2:1 initial assessment 6 min walk test and given a score of perceived fitness and confidence by HP *Class* (15 min warm up; 20 min stations (2×10 min); 10 min cool down) Relaxation session once a week Weekly information seminars
WHO	Cardiac PT Physiotherapy assistant CR coordinator	Cardiac physiotherapist Cardiac specialist Nurse Additional PT ×2	Specialist physiotherapist 2 CR nurses
HOW	Group classes 15–20 per class Hospital gym Low level classes available	Group classes 15–25 per class Main district hospital AND Local community sports centre Low level classes available	Group classes (maximum 15 patients) Leisure centre Dance studio facilities
WHERE	Hospital gym	Hospital or sports centre	Sports centre
WHEN and HOW MUCH (dose)	*Frequency*: once a week for 10 weeks (10 sessions) *Intensity*: 12–14 RPE (Borg 6–20 RPE scale) *Time*: 75 min sessions (50 min exercise component) *Type*: Both CV and resistance/strength stations	*Frequency*: once or twice a week for 12 weeks *Intensity*: 3–4 RPE (Borg CR10 Scale). ‘Talk test’ also used. Observation from healthcare team *Time*: 90 min (55 exercise component) *Type*: both CV and resistance/strength stations	*Frequency*: twice a week for 6 weeks (12 sessions) *Intensity*: RPE and HR monitor given, with patient-specific ranges to work within *Time*: 75 min (50 min exercise component) Type: both CV and resistance/strength stations

CR, cardiac rehabilitation; ISWT, Incremental Shuttle Walk Test; PT, physiotherapist; TIDieR, Template for Intervention Description and Replication.

Additionally, patients with CRC were invited along to the education sessions delivered by the CR team. Session themes across the three sites included healthy lifestyle sessions (eg, diet, physical activity, relaxation/stress management), and cardiac-specific sessions (eg, misconceptions, medications, ‘healthy heart’). Cancer-specific sessions were not provided, as this was not possible across the three sites.

*Training*: CR physiotherapists and other CR healthcare professionals received training about cancer and exercise before any patients with CRC were referred to CR. Training was delivered by a cancer and exercise specialist (AC) in 1 day, face-to-face in sites 1 and 2 and by video conferencing in site 3. Training covered evidence of the benefits of exercise, principles and guidelines of exercise prescription contraindications, and red flags and issues to monitor before and during exercise programme. Practical examples of circuit-based exercises, working at different levels of intensity, principles of exercise motivation and facilitating health behaviour change were demonstrated.

#### Outcomes

Trial outcomes used to assess the feasibility and acceptability of the intervention were consent rate, reasons for declining to participate, adverse events, intervention adherence, evaluation of training.

#### Sample size

The aim of the study was not to provide a definitive estimate of treatment effect, so we did not have a formal sample size calculation. Rather, we estimated that we would recruit 66 participants in a given time period.

#### Data collection

In this manuscript, we describe data collection for parameters that directly address the feasibility and acceptability of the intervention.

*Consent rate* was calculated by dividing the number of people with CRC who met inclusion criteria and therefore eligible, by the number who consented to participate in the study.

*Reasons for declining to participate* were recorded by site investigators.

*Adverse events:* If a participant experienced an adverse event (eg, death, inpatient hospitalisation or prolongation of existing hospitalisation, persistent or significant disability/incapacity) during the course of study, an adverse event report was completed. Any adverse event considered to be ‘related’ or ‘unexpected’ (eg, twisted ankle) was reported to the NHS research ethics committee and the study sponsor.

*Intervention adherence and attendance* was measured by the total number of planned CR classes attended by participants allocated to the intervention group. Data were collected from the CR register of attendance.

*Cancer and exercise training* was evaluated by participants completing an evaluation form, which included 18 questions covering precourse information, course content, course venue and facilities. Questions were a combination of scaled questions 1–5 (strongly agree 5; strongly disagree 1) and open text questions.

#### Analysis

Descriptive statistics were used to summarise the consent rate, reasons for declining to participate, adverse event and intervention adherence rates and the findings of the evaluation of cancer and exercise training.

### Qualitative study

Thematic analysis—“A method for identifying, analyzing and reporting patterns within data”^25^ was the methodological framework underpinning the qualitative study that was nested within the pilot RCT.

#### Participant selection

Purposive sampling was used to select people for participation in the qualitative study as follows:

*CRC survivors*: All trial participants (randomised to intervention or control groups) were contacted by telephone and invited for interview.

*People with CVD*: In each site, at one CR class, all people with CVD were invited by a CR clinician to attend a focus group at a specific time and day.

*Healthcare professionals*: All CRC nurses involved in recruitment and CR physiotherapists and nurses delivering the intervention (ie, CR) were invited to attend a semistructured face-to-face interview at the end of the intervention delivery period.

#### Data collection

Interview and focus group schedules were used to assist the investigator in gathering responses about the feasibility and acceptability of the intervention. Table 2 summarises the key topic areas explored with each group relating to intervention feasibility and acceptability. With participants’ permission interviews and focus groups were audio-recorded.

**Table 2:** Key topic areas explored relating to intervention feasibility and acceptability

	Health professionals	Patients with CRC	CVD groups
Barriers			
Travel/distance	✓	✓	✓
Recovery from surgery	✓	✓	✓
Stoma	✓	✓	
Adjuvant therapy	✓	✓	
CR as part of routine care	✓	✓	✓
Mixed patient classes	✓	✓	✓
Capability of group	✓		✓
Capacity of services	✓	✓	✓
Gaps in support	✓	✓	✓
Reasons for taking part		✓	
Randomisation process		✓	
Study information		✓	
Data collection	✓	✓	

CR, cardiac rehabilitation; CRC, colorectal cancer; CVD, cardiovascular disease.

#### Analysis

Two investigators (GH and JM) analysed qualitative data. Audio-recorded interviews/focus groups were transcribed verbatim and analysed thematically. The Framework approach, which is a rigorous method providing a structure within which qualitative data are organised, coded and themes identified, was used to guide the analysis.^26^ ^27^

### Ethical approval and research governance

NHS ethics approval was provided (REC reference 13/NS/0004; IRAS project ID 121757). NHS Research Management approvals (an additional approval required in the UK for research involving NHS patients, staff or premises) were provided by each of the three Health Boards where the study was conducted.

## Results

### Consent rate

Seventy-four out of 133 (55.6%) eligible CRC survivors indicated that they were interested in participating in the study. Thirty-one per cent (n=41) consented to participate in the study.

### Reasons for declining to participate

Table 3 shows reasons for declining to participate in the study. The most common reason why those interested in participating withdrew before formally consenting to the study fell into the clinical category (9 out of 33, 27%), including recovery from surgery, poor mobility and comorbidities. We also had 18% (6 out of 33) of patients unable to attend while receiving their adjuvant therapy following surgery, due to tiredness and fatigue. In total, these factors accounted for 45% of all declining patients.

**Table 3:** Reasons for declining to participate (n=33)

Reason	All sites
Distance/travel barriers	2 (6%)
Return to normal activities	3 (9%)
Clinical, for example, poor recovery from surgery, comorbidity	9 (28%)
Other commitments/time	2 (6%)
Adjuvant therapy	6 (18%)
Study time limit	3 (9%)
Unable to contact	1 (3%)
Patient death	1 (3%)
Missing	6 (18%)

### Adverse events

No adverse events were reported.

### Adherence and attendance

Thirteen out of 21 participants (62%) completed the 10/12 week CR programme. Three participants started CR but could not complete all CR classes and five did not begin CR (38%). The main barrier to not starting or dropping out of CR was poor health (n=7), musculoskeletal issues (n=2), further surgery, uncontrolled hypertension, mental health issue, chemotherapy side effects (n=2). Participants who were able to continue CR had high levels of attendance (range 75–142%), with four participants attending additional classes. Further details by site are illustrated in table 4.

**Table 4:** Adherence and attendance by site

	Number of sessions	Adherence (%)	Attendance (%)
Site 1	1/week for 10 weeks=10	83	100
Site 2	1–2 weeks for 12 weeks=12–24	56	107
Site 3	2 weeks for 12 weeks=12	50	92

### Cancer and exercise training evaluation

Twenty health professionals (10 CR physiotherapists/assistants and four cardiac nurses six CRC nurses) were trained. Fourteen (70%) evaluation forms from across all three sites were completed and returned; 6 (30%) forms were not returned. All 18 scaled questions marked highly with a score of 4 or 5—with 5 being the maximum possible score. Attendees, for instance, reported that the course content was at the appropriate level, and was well presented, and all said they would recommend the course to a colleague.

### Qualitative findings

Forty-one participants were involved in the qualitative study including 22 CRC survivors (12 intervention, 10 control), 8 people with CVD, 5 CRC nurses and 6 CR clinicians. Thus, just over half of all CRC survivors participating in the pilot RCT participated in the embedded qualitative study. All CRC nurses involved in screening for eligibility and all CR physiotherapists delivering classes with CR survivors participated in the qualitative study. Themes are described and a quotation to illustrate each theme is presented. For all quotations, letters followed by a unique number are used as participant identifiers, for example, letters indicate the following: CR: Cardiac Rehabilitation clinician, CRC nurse: Colorectal cancer nurse, CRC survivor, CVD: patient with cardiovascular disease. The groups in which CRC survivors participated are indicated by ‘intervention’ or ‘control’.

#### Confidence and motivation

CR was perceived to give CRC survivors the confidence to start to become more physically active following CRC diagnosis and treatment.

*Investigator*: So what did you get out of it the most do you think?

*Participant*: Confidence probably.

*Investigator*: Confidence that you could exercise?

*Participant*: Yes, yes. (CRC survivor 30 intervention)

CR provided a structure and regular opportunity to exercise, which was believed to motivate people to engage in physical activity.

I'd be confident but just not motivated so I need somebody to give me a kick up the butt and say ‘Come on you've got to do this’ and I will do it.’(CRC survivor 02 control)

#### Peer support

CR was a social opportunity where people could tap into support from their peers as well as an exercise opportunity.

And we all fell into the same trap: ‘Oh, did you do your exercises?’ ‘What do you mean, since last week?’ and, ‘Oh yes, last night,’ you know[laughs]. But and then it got better, I got a bit more disciplined about it. But I’ve, an important point here, which is the companionship during the sessions, but also before the sessions, because we were encouraged to meet sort of ten minutes before the class so we were all there on time. (CRC survivor 16 intervention)

#### Mixed classes

None of the participants (ie, people with CRC or CVD or clinicians) had a problem with people with a different condition (cancer) attending CR.

*Investigator*: So what are your initial thoughts when I say, “putting cancer patients in your cardiac class”?

*P1*: I don't see why not, and if they're just the same, why not?

*P2*: Yeah.

*P3*: The facilities can take it, I don't see why not. (CVD 02)

#### Support from health professionals

CR professionals emphasised that a key advantage for people attending CR was the quality of support they would receive if NHS health professionals, able to offer them a greater degree of safety and understanding of their illness experiences, delivered it.

I think the thing that sold it was the fact that there was going to be physiotherapists and nursing staff there with the patients because they worry about hurting themselves and they were all quite happy to do whatever as long as they were under supervision and I, I got that from all the patients I spoke to. They would not have gone into a gym without something knowing what they had been through. And it gave them re-assurance from them and that's why some of them took it on when they were people who may be did exercise anyway because they were worried about the wound and the work that had been done inside and so that, that was definitely a bonus. (CRC nurse 007)

#### Barriers to CR

Travel distance acted as a barrier for attending CR.

It can be difficult because this area covers, its wide you know it's a huge distance for a lot of people to travel, so for some patients it is, it is a problem and we've had cardiac patients that won't come because transport is a problem. (CR 002)

There were, however, some barriers and concerns that were seen to be unique for people with CRC, which were protracted recoveries from abdominal surgery, chemotherapy and stoma.

There were some CRC patients who were fit and then something would happen to them and they basically crashed may be a couple of may be a week or two after surgery…Because they had kind of side-effects and wound infections and chest infection problems that erm it took may be months actually to get over. (CRC nurse 007)

#### Capability

CR professionals were concerned that they would not have the relevant knowledge and skills to support people with cancer since their specialism was cardiology.

They've[people with CRC] obviously got different issues from our cardiac patients and what we're finding is that they got a lot of psychological issues now that we're having to deal with, whereas it probably would have been more relevant for, you know a specialist nurse in that area or possibly a physiotherapist in that area that probably could deal with their problems slightly better…we've got very minimum skills to do that. (CR 003)

Nevertheless, CR professionals recognised that the exercise component of CR was generic to people, regardless of their specific condition. Indeed, exercise was individually tailored by fitness level and not by the type of disease that a person was recovering from.

*Researcher:* Did you tailor the classes for our patients?

*CR:* No not at all. Absolutely no difference whatsoever in the class. We tailor the exercises individually but not because they were cancer patients. (CR 004)

#### Capacity

Alongside voicing concerns regarding their own capabilities to support people with CRC, CR professionals were also concerned about the capacity of CR to accommodate more patients.

Whether it would affect the numbers in the classes, whether we would have to run extra classes and whether my waiting lists would go up. (CR 001)

#### Education sessions

CR includes exercise and information sessions. CR professionals believed that some of the information sessions would be relevant to people with CRC as well as people with CVD. These included sessions about the benefits of exercise, stress management, relaxation and healthy lifestyle. CR professionals reported that people with CRC attended most information sessions. Nevertheless, CR professionals noted that they were unable to provide some specialist information for people with CRC due to the information sessions being geared towards people with CVD.

We obviously offer dietetic input and a lot of the bowel cancer patients were interested in the dietetic side of things but they were having issues with the dietician because although its general healthy living, they feel that they need specific dietary advice…so that was you know a gap that you're sort of noticing with the service. Its may be that you know they might need some sort of more dietary input as well to see what they can and cannot eat and what would be beneficial for them. (CR 003)

## Discussion

Bowen *et al*^28^ recommend eight areas of focus to assess if a public health intervention is feasible. Addressing each area can help inform assessment of the feasibility and acceptability of CR for postsurgical CRC survivors and the likelihood of this model of rehabilitation being implemented as part of routine cancer care and as a future commissioned service. These eight areas are discussed in light of the study findings and in relation to other literature.

### Acceptability

To what extent is a new idea, program, process or measure judged as suitable, satisfying, or attractive to program deliverers? To program recipients?

It is possible to run mixed CR classes for CRC survivors and people with CVD. Indeed, CRC survivors believed that a benefit of rehabilitation was peer support with support coming from people with CVD as well as other CRC survivors attending CR. Traditionally, peer support has been defined as support provided by people with the same disease.^29^ Shared experience of the disease and experiential empathy is seen as crucial to the giving and receiving of support.^29^ ^30^ This study challenges the assumption that peer support for people with cancer can only arise from shared experience of the same disease.^31^ Rather, our study suggests that people with CRC can obtain peer support from people with CVD in the context of rehabilitation. That peer support is not disease-dependent, opens up the possibilities of rehabilitation for mixed disease patient groups. Moreover, our study raises the prospect of redefining peer support, so that it is not exclusively confined to shared experience of a specific disease; a finding also noted by a recent review of self-management support interventions for men with long-term conditions.^32^

### Demand

To what extent is a new idea, program, process, or measure likely to be used (i.e., how much demand is likely to exist?)

If the consent rate is a proxy for level of demand by CRC survivors for CR if it were to be implemented in practice, then based on this trial, 31% of CRC survivors are likely to take up the offer of CR should this service be offered to them. This would mean that demand by CRC survivors would be 12% less than the number of people with CVD who attended CR in 2011–2012 (43%) in the UK.^33^ Given that CR for people with CVD is a well-established service that has been audited by the British Heart Foundation (a UK charity) since 2004, a rate of 31% engenders optimism that up-take among CRC survivors would eventually match attendance rates among people with CVD. Other physical activity intervention trials report recruitment rates ranging from 8% to 98%,^34–39^ suggesting that demand for physical activity interventions by CRC survivors are highly variable and appear to be unrelated to intervention mode (ie, counselling, home-based exercise prescription, exercise classes). Our study found barriers to participation were protracted recoveries from surgery and ongoing treatments. Other studies have also reported medical conditions as a reason why eligible participants do not participate.^36^ ^38^ ^40^ These barriers are likely to impact demand on rehabilitation. Nevertheless, we acknowledge that recruitment rates and barriers for involvement in research and patient use of an actual service are not directly comparable.

Motivation is a key construct in theories of behaviour change and has been associated with higher levels of physical activity among CRC survivors.^41^ According to self-determination theory, internalisation of the value (the benefits) of the outcomes of physical activity is likely to lead to greater persistence in being physically active.^42^ Demand for a physical activity intervention such as CR is therefore likely to increase as the benefits of physical activity for CRC survivors become more widely known. Recent studies, however, indicate that provision of lifestyle advice, including the benefits of physical activity is low,^43–46^ which suggests that demand for CR (and indeed other physical activity interventions) may remain suboptimal until evidence of the benefits of physical activity are conveyed to CRC survivors. Educational efforts on the benefits of PA for patients with CRC have the potential to improve demand and uptake of this type of intervention. The evidence is strong^5–10^ and growing, and demand is likely to continue to increase, as health professionals and patients alike become aware of that.

### Implementation

To what extent can a new idea, program, process, or measure be successfully delivered to intended participants in some defined, but not fully controlled, context?

This was a pragmatic trial and a major strength and advantage of pragmatic trials is the testing of already existing services in real-world settings. It is very different therefore to an explanatory trial where the intervention is tightly controlled and managed by the investigating team. Pragmatic trials therefore provide relatively strong evidence about the potential for implementation. To the best of our knowledge, there are few pragmatic trials of a physical activity intervention for people with CRC.

The study suggests that CR physiotherapists can receive additional training in cancer and exercise and that they can support CRC survivors to exercise safely. Indeed, no adverse events were reported during the trial, suggesting that CR for CRC survivors is safe. Moreover, the qualitative study suggests that postsurgical CRC survivors welcome support to increase their level of physical activity from trained healthcare professionals. Thus, CR physiotherapists may be a particularly appropriate group of professionals to deliver a physical activity intervention to cancer survivors. In the UK, physiotherapists are registered with the Health and Care Professions Council (HCPC) and will have successfully completed a HCPC-approved programme in physiotherapy (offered as 3-year or 4-year undergraduate degrees and 2-year postgraduate levels at various UK universities). The training involves both periods of theory and clinical experience gained by meeting and working with patients. The theory part of the course covers anatomy, physiology, physics and pathology. CR physiotherapists are experienced in prescribing exercise for patients with a range of conditions.

Intervention adherence refers the extent to which participants randomised to the intervention group follow specific treatment therapy instruction as per intervention protocol and can therefore be a useful proxy for implementation. The study suggests that two-thirds of CRC survivors will complete a 12-week CR programme, and the main reason CRC survivors are unable to start or stop attending CR will be poor physical health. Nevertheless, CR attendance by CRC survivors who are able to partake in the intervention is likely to be high. Other trials also report high levels of adherence,^35–40^ ^47–49^ suggesting that physical activity interventions for CRC survivors can be successfully delivered.

### Practicality

To what extent can an idea, program, process, or measure be carried out with intended participants using existing means, resources, and circumstances and without outside intervention?

The qualitative study suggests that there are concerns about CR capacity should this service be offered to CRC survivors. It is likely therefore that were this service to be offered to CRC survivors then additional resources such as employment of additional staff (eg, a physiotherapy assistant) would be required. We anticipate that the overall additional costs are likely to be modest; for instance, the overall cost for of an 8-week, physiotherapy-led exercise intervention in deconditioned cancer survivors in the early survivorship period (the PEACH trial) conducted in Ireland was €196 per participant, including the salaries of the clinicians, overheads and equipment costs.^50^

### Adaptation

To what extent does an existing idea, program, process, or measure perform when changes are made for a new format or with a different population?

The study suggests that existing CR can perform with a different population (ie, CRC survivors) and that physiotherapists do not need to adapt the exercise class to support CRC survivors. The study suggests that exercise prescriptions are for individuals and not the disease per se. Indeed, the American College of Sports Medicine (ACSM)^51^ expert panel's recommendations for aerobic, resistance and flexibility exercises for cancer survivors are the same as the age-appropriate physical activity guidelines for the general population with several alterations if required. ACSM made it clear that medical assessment prior to beginning physical activity is not required and may act as a barrier to engaging in physical activity.^51^

### Integration

To what extent can a new idea, program, process, or measure be integrated within an existing system?

This study suggests that referral pathways can be introduced, so that CRC nurses refer CRC survivors to CR. In addition, CRC nurses can provide information (eg, type of treatment, medication, comorbidities) about patients with CRC to the CR team, so that they can support people with CRC to exercise safely. Given that multidisciplinary teams are emerging in cancer care,^52^ ^53^ the notion of integrating CR within existing cancer service pathways may increasingly become acceptable.

### Expansion

To what extent can a previously tested program, process, approach, or system be expanded to provide a new program or service?

A comparison of studies of coronary heart disease and the post-treatment needs of CRC survivors suggests that there is reasonable justification for expanding CR to include CRC survivors. Four qualitative studies of patients’ experiences of needs after coronary artery bypass grafting^54–57^ and a case note review of needs of 521 patients surgically treated for CRC cancer^58^ and a population-based cohort study including 522 people with CRC^59^ indicate that people with CVD and people diagnosed with CRC experience similar problems including pain, fatigue, anxiety and depression, worry, appetite loss, sexual problems, sleep disturbance, and work and financial-related difficulties and express a need for information about medication and self-management. Thus, the rehabilitation needs of people with CVD and CRC survivors are likely to be similar, suggesting that a common rehabilitation programme may be appropriate. Moreover, CR may be particularly relevant for people with CRC since the estimated prevalence of CVD is 59% at 5 months postdiagnosis and 16% develop de novo CVD within 36 months after treatment.^60^ In addition, common comorbid conditions in CRC survivors include congestive heart failure, diabetes mellitus and chronic obstructive pulmonary disease,^61^ which again may be managed by rehabilitation.

Pointing out the similarities in post-treatment experiences is not to deny that there are, of course, disease-related differences among different patient groups. For example, CRC survivors can experience physical discomfort and bowel function problems and urinary tract infections and need advice about abdominal pain and stoma care,^62^ which are almost certainly likely to be problems that are not experienced by those with CVD unless they have comorbidities. The study, however, suggests that CR physiotherapists did not feel competent providing specific CRC-related advice and support such as stoma care. Rather than expanding CR to provide CRC-specific advice and support, the existing cancer care team could continue to provide cancer support to address CRC survivors’ cancer-related needs, but there would need to be closer links between CR and cancer care.

### Limited efficacy

Does the new idea, program, process, or measure show promise of being successful with the intended population, even in a highly controlled setting?

We did evaluate outcomes but focused on examining the feasibility and acceptability of the intervention. The trial shows that poor health is a barrier to participating; yet, these CRC survivors may also benefit from rehabilitation, perhaps even more so than their healthier counterparts? Other studies have also reported medical condition and ongoing treatment as reasons why eligible CRC survivors did not participate.^36^ ^38^ ^40^ Thus, the success of a future trial of the CR for CRC survivors may depend on the extent to which those in poorer health and consequently in greatest need of rehabilitation participate.

### Strength and limitations

A key strength of this study lies in its purpose to test feasibility and acceptability in a pragmatic pilot trial with embedded qualitative study prior to undertaking any large-scale, costly future trial of the intervention. The decision to explore the acceptability and feasibility of the CR model—an already evidence-based and established rehabilitation model—for people with cancer is significant within the context of the current healthcare climate and the need for effective resource use and cost savings wherever possible. It is possible, however, that some of the perceptions of CR for postsurgical CRC survivors presented here represent a select cohort who were already motivated to, and interested in, being part of a physical activity intervention They may, for example, already have held positive views towards behaviour change, and in particular change in physical activity as a core component of their recovery. In addition, the interviews were conducted by the investigators involved in collecting baseline and follow-up measures from CRC survivors, which may have influenced the extent to which participants were willing to be critical. Nevertheless, these investigators were not involved in the direct care of participants and in particular, they were not involved in delivering the intervention (ie, CR) and so participants may have been more candid about their views of the intervention itself. The generalisability of our findings, however, is limited because the pilot was small scale involving only 3 out of a possible 312 CR programmes throughout the UK^48^ ^49^ and involving only small numbers of CR and CRC healthcare professionals and people with CRC and CVD. The findings, nonetheless, provide valuable insights and a starting point for informing future healthcare.

### Implications for CRC survivors

We can be confident that CR is an acceptable and feasible rehabilitation service for postsurgical CRC survivors and their clinical care teams. The aim of this pragmatic trial was not to attempt to change and adapt CR but to find out if it is feasible and acceptable to refer people with CRC to this current service as it is currently configured. However, before we recommend UK-wide implementation, it is critical that some of the key barriers identified in this study are addressed and whether this model of rehabilitation has a health benefit for people with cancer.
